# 16HBE Cell Lipid Mediator Responses to Mono and Co-Infections with Respiratory Pathogens

**DOI:** 10.3390/metabo10030113

**Published:** 2020-03-18

**Authors:** Daniel Schultz, Surabhi Surabhi, Nicolas Stelling, Michael Rothe, KoInfekt Study Group, Karen Methling, Sven Hammerschmidt, Nikolai Siemens, Michael Lalk

**Affiliations:** 1 Institute of Biochemistry, University of Greifswald, 17487 Greifswald, Germany; daniel.schultz@uni-greifswald.de (D.S.); methling@uni-greifswald.de (K.M.); 2 Department of Molecular Genetics and Infection Biology, University of Greifswald, 17487 Greifswald, Germany; surabhi.surabhi@uni-greifswald.de (S.S.); nicolas.stelling@uni-greifswald.de (N.S.); sven.hammerschmidt@uni-greifswald.de (S.H.); 3 Lipidomix, 13125 Berlin, Germany; michael.rothe@lipidomix.de

**Keywords:** eicosanoids, lipid mediators, oxylipins, respiratory tract infection, co-infection, *S. aureus*, *S. pneumoniae*, influenza A virus

## Abstract

Respiratory tract infections are a global health problem. The main causative agents of these infections are influenza A virus (IAV), *Staphylococcus aureus* (*S. aureus*), and *Streptococcus pneumoniae* (*S. pneumoniae*). Major research focuses on genetics and immune responses in these infections. Eicosanoids and other oxylipins are host-derived lipid mediators that play an important role in the activation and resolution of inflammation. In this study, we assess, for the first time, the different intracellular profiles of these bioactive lipid mediators during *S. aureus* LUG2012, *S. pneumoniae* TIGR4, IAV, and corresponding viral and bacterial co-infections of 16HBE cells. We observed a multitude of altered lipid mediators. Changes in the amount of 5-hydroxyeicosatetraenoic acid (5-HETE) were prominent for all bacterial infections. The infection with *S. pneumoniae* showed the strongest impact on bioactive lipid production and led to alterations in the amount of PPARγ ligands and precursors of pro-resolving lipid mediators.

## 1. Introduction

Infections of the respiratory tract are the fourth most common reason for the cause of mortality worldwide [1]. Major pathogens causing such infections are influenza A virus (IAV) [2], *Staphyloccocus aureus* (*S. aureus*), including methicillin-resistant strains of the USA300 lineage, and *Streptococcus pneumoniae* (*S. pneumoniae*) [3]. There is evidence that bacterial and viral co-infections even accelerate the mortality rate as compared to infections by single agents [4]. The immune system plays a major role in the recognition and elimination of the pathogens, which can be influenced by bioactive lipids. Such bioactive lipid mediators are eicosanoids. They play a role in, e.g., induction and resolution of inflammation [5,6,7]. These oxidized unsaturated fatty acids are synthesized from arachidonic acid (AA), docosahexaenoic acid (DHA), eicosapentaenoic acid (EPA), and linoleic acid (LA) [8]. The main enzymes responsible for the biosynthesis of these oxylipins are lipoxygenases (LOX), cyclooxygenases (COX), and cytochrome P450 enzymes (CYP). AA conversion by LOX results in the production of hydroxyeicosatetraenoic acids (HETEs) or hydroxydocosahexaenoic acids (HDHAs) with DHA as substrate. CYP are responsible for the production of epoxyeicosatrienoic acids (EETs) and hydroxyeicosapentaenoic acids (HEPEs). The main products of COX are prostaglandins. For a review, see [8,9,10,11]. Several immuno-supportive properties are described for eicosanoids and other lipid mediators. These includes vasomodulation [12], chemoattraction [13], and G protein-dependent secretion of interleukin (IL)-6 and IL-8 [14]. Anti-inflammatory eicosanoids (e.g., 12-HETE, 15-HETE), mainly block immune cell infiltration [15], inhibit secretion of interleukins [16] or activate the peroxisome proliferator-activated receptor (PPAR) on immune cells [17]. Most research on eicosanoids is focused on chronic diseases, including asthma or inflammatory bowel diseases [18,19]. However, the majority of research on host eicosanoid profiles in infectious diseases is restricted to single infections with the pathogens *Escherichia coli* [20], *Borrelia burgdorferi* [21], *Pseudomonas aeruginosa* [22], and IAV [5,23,24] or focused solely on prostaglandin E_2_ [25,26,27,28] and its related receptors [28]. 

To our knowledge, lipid mediator profiling in bacterial, in particular *S. pneumoniae* and viral co-infections, is unexplored (Table 1). Here, we used human bronchial epithelial cell line 16HBE [29,30,31] for single and co-infections with IAV, *S. aureus,* and *S. pneumoniae*. Epithelial cells are the initial protective barrier against viruses and bacteria and an important lung compartment to initiate immune system regulation. It is well documented that IAV-mediated lung tissue damage increases the susceptibility of the human host to secondary bacterial infections [32,33]. The analysis of the pathogen-mediated changes in the intracellular lipid mediator profile could help to understand pathogen-specific host immune responses in viral and bacterial single as well as co-infections.

## 2. Results

### 2.1. Viability of 16HBE Post Infections

Most infections led to a minor, insignificant drop in cell viability (Figure S1). These included IAV rH1N1 and *S. pneumoniae* TIGR4 single and respective co-infection. However, the strongest effects were observed for *S. aureus* LUG2012 in single and co-infection with IAV rH1N1. The amount of vital cells dropped down by approximately 50% (Figure S1).

### 2.2. Intracellular Lipid Mediator Profile in Response to Bacterial and Viral Single and Co-Infections

**Figure 1:**
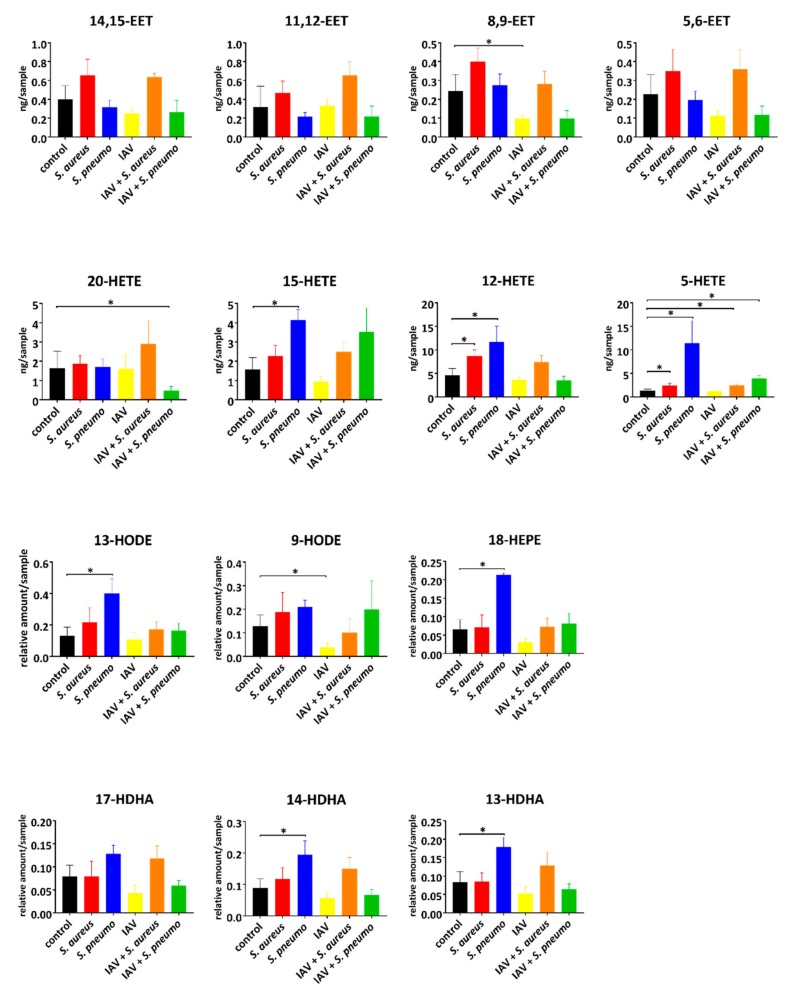
Intracellular lipid mediator amounts in response to indicated bacterial and viral infections: *S. aureus* LUG2012 (red), *S. pneumoniae* TIGR4 (blue), IAV rH1N1 (yellow), uninfected control (black) and the co-infections IAV/*S. aureus* (orange), as well as IAV/*S. pneumoniae* (green). The bars denote mean values ± standard deviations. For statistical analysis, the Mann–Whitney U-test was used for *n* = 15 (controls) and *n* = 4 (infections). The *p*-values were compared to a significance level α of 0.05 corrected for multiple comparisons using Bonferroni correction for 9 clusters of oxylipins and 5 comparisons resulting from infection conditions. Asterisks indicate significant changes.

For all indicated infection conditions, we were able to detect 14 eicosanoids and oxylipins (Figure 1 and Table 1) out of 21 lipid mediators via our MRM method (Table S1). In general, a mixed profile of AA, DHA, EPA, and LA derived lipid mediators was observed. 

Eicosanoids from AA conversion (HETEs) showed changes for all tested infections, whereas DHA- and EPA-derived and analyzed lipids (HDHAs and 18-HEPE) were only affected by single infection with *S. pneumoniae* TIGR4. In particular, the pro-inflammatory 5-HETE was elevated in all bacterial infections, including co-infections (Figure 1). We detected a basal level of approximately 1.4 ng/sample (control), which was significantly increased in all single bacterial and bacterial and viral co-infections. The IAV single infection itself had no impact on 5-HETE production (Figure S2). In addition, increased levels of 12-HETE were detected in both staphylococcal and pneumococcal mono infections.

The majority of changes in the lipid mediator profiles were detected in *S. pneumoniae* infections. Enhanced amounts of 15-HETE, 13-HODE, 18-HEPE, 13-HDHA, and 14-HDHA were exclusively detected in pneumococcal infections (Figure 1 and Figure S2). Both anti-inflammatory lipids 13-HODE and 18-HEPE showed high levels (fold change >3) under infection conditions compared to control. In IAV single infections, only suppressive effects on the amounts of 8,9-EET and 9-HODE were observed. The production of 9-HODE can be derived by enzymatic and autoxidation processes [34]. Moreover, decreased levels of 20-HETE were detected in pneumococcal and IAV co-infections (Figure 1).

## 3. Discussion

Lipid mediator profiling of infected 16HBE cells revealed that 5-HETE might play a major role at initial steps of bacterial infections of the respiratory tract. 5-HETE levels were significantly increased in 16HBE cells infected with *S. aureus* LUG2012, *S. pneumoniae* TIGR4, and during the corresponding co-infections with bacteria and IAV rH1N1, respectively. This observation may indicate a prominent role of the pro-inflammatory metabolite 5-HETE in bacterial infections of the bronchial compartment. 5-HETE is known to enable transcellular migration and aggregation of neutrophils and to induce airway contraction [35,36]. This lipid mediator is generated from AA within the 5-lipoxygenase (LO) pathway, encoded by the *ALOX5* gene [37]. The interplay of 5-LO with the nuclear membrane-associated 5-LO activating protein FLAP results in AA oxidation to 5-hydroxyperoxyeicosatrienoic acid (5-HpHETE). Subsequently, 5-HpHETE is than converted by glutathione peroxidase to 5-HETE or to leukotrienes. Moreover, the different pro-inflammatory 5-LO eicosanoids are associated with diseases like diabetes [38], Alzheimer [39], and atherosclerosis [40]. For example, leukotriene A_4_ can be converted to the pro-inflammatory leukotriene B_4_. Clementsen et al. observed enhanced amounts of leukotriene B_4_ by antibody based detection in the supernatants of human bronchoalveolar cells treated with inactivated *S. aureus*. However, this was not seen in IAV-infected cells [41]. This indicates that, besides the pathogenic bacteria itself, toxins or bacterial surface structures may also lead to alterations in eicosanoid production. Indeed, lipopolysaccharide of Gram-negative bacteria is able to induce increased production of prostaglandins in endothelial cells [42]. Furthermore, no changes in the 5-HETE production in response to IAV rH1N1 single infection were observed. This is in line with other studies showing that IAV infections of mice [5,23], as well as pigs [24], do not show changes for this lipid mediator, suggesting a lack of 5-LO activation by IAV. In the pig IAV infection study [24], whole organs and BAL-fluid did not discriminate between different cellular compartments, which can be obtained by single-cell experiments. However, a study by Tam and colleagues showed increased levels of 5-HETE in nasopharyngeal washes of human high responders during the 2009-2011 influenza seasons [5]. Whether such responses are linked to a certain human organ, cellular compartment or host genetics remains to be elucidated. In contrast to other IAV infection studies [5,23] using high pathogenic IAV strains, we used the rH1N1 (A/Bavaria/74/2009), which is non-high pathogenic and more appropriate for co-infections. This could be a possible reason for the missing 5-LO activation by IAV. However, the interplay between IAV and secondary bacterial infection is complex. Thus it remains unclear how viral virulence and co-infection affect 5-HETE production in the whole human lung, even if there are some suggestions that higher virulent IAV strains increase rates of viral pneumonia and secondary bacterial infections together with associated mortality [4,43,44].

Furthermore, we show that *S. pneumoniae* TIGR4 infections have the strongest impact on the oxylipin profile. Induced 15-HETE and 13-HODE levels were exclusively seen in these infections. Both 15-HETE and 13-HODE are PPARγ agonists. PPARγ is a ligand-activated transcription factor, and it is known for its anti-inflammatory properties. These include downregulation of the cyclooxygenase-2 gene [45], inhibition of NF-κB [46], or reduced production of pro-inflammatory cytokines and interleukins through its action on macrophages [47]. The group of Solleti et al. [48] showed that the loss of epithelial PPARγ in the lung leads to increased inflammatory mediator production. This could explain why enhanced PPARγ activation is necessary for the resolution of inflammation. 

Furthermore, we observed decreased amounts of 20-HETE in co-infections with *S. pneumoniae*. A relationship between changes in levels of 20-HETE and immune suppression in the context of immunosuppressant medication is described [49]. Nevertheless, the role of this lipid mediator in the context of inflammation is rarely explored.

In line with this, elevated amounts of other anti-inflammatory lipid mediators (12-HETE or 18-HEPE) were also detected in pneumococcal infected 16HBE cells. The simultaneous increase of both oxylipins with pro-inflammatory (5-HETE) and anti-inflammatory properties (15-HETE and 13-HDHA) was also documented in IAV-mediated respiratory tract infections of mice [23]. However, our study shows, for the first time, such a mixed profile in *S. pneumoniae* infections. For further studies, infection experiments that are closer to in vivo conditions of tissues like air–liquid interface cell culture should be taken into account for verification of our findings. 

## 4. Materials and Methods 

### 4.1. Chemicals

Lipid mediators and the deuterated standards 12-HETE-d8 and 13-HODE-d4 were purchased from Cayman chemicals. 12-HETE-d8 and 13-HODE-d4 were dissolved in acetonitrile (both 100 ng/ml as internal standard) on ice and aliquots were stored at −80 °C until usage. Acetonitrile (MS grade) was purchased from Th. Geyer^®^, methanol from Roth^®^, and acetic acid (HPLC grade) from VWR^®^. Solid-phase extraction cartridges Bond Elut Certify II (3 mL, 200 mg) were obtained from Agilent^®^. BHT and other chemicals, including hexane, ethyl acetate, and sodium hydroxide were purchased from Sigma-Aldrich.

### 4.2. Cell Culture

16HBE14o- (16HBE) cells were a gift from Dieter Gruenert (Mt Zion, Cancer Center, San Francisco, CA, USA). The cells were cultured in MEM medium (Gibco) supplemented with 10% (*v*/*v*) fetal bovine serum (FBS; Life Technologies), 2 mM L-glutamine (Invitrogen), 10 mM HEPES (GE Healthcare) and 1% (*v*/*v*) Minimal Essential Amino Acids (GE Healthcare) in fibronectin-coated flasks (Corning) at 37 °C and 5% CO_2_ atmosphere.

### 4.3. Bacterial and Virus Strains

*Streptococcus pneumoniae* serotype 4 strain TIGR4 was grown on Columbia blood agar plates (Oxoid) and cultivated to mid-log phase (A_600_ 0.35–0.4) in Todd–Hewitt broth (Roth) containing 0.5% (*w/v*) yeast extract (Roth) at 37 °C and 5 % CO_2_. *Staphylococcus aureus* strain LUG2012 (USA300 lineage) [50] was cultured overnight at 37 °C in casein hydrolysate and yeast extract (CCY) medium with agitation [51]. Influenza virus A/Bavaria/74/2009 (rH1N1) was propagated and cultivated, as described by Eisfeld and colleagues [52].

### 4.4. Cell Culture Infections Experiments

16HBE cells were seeded in T175 culture flasks and grown to approximately 80% confluence. For calculation of the multiplicity of infection (MOI), cell counts of the control flask were determined. The flasks were either left untreated or were infected with rH1N1 at MOI 0.1 for 24 h. This amount of virus and the time point were determined as an optimal infection with no significant cell death. After 24 h, bacterial infections of uninfected and rH1N1 infected cells were performed. TIGR4 infections were conducted at MOI 50 for 2 h, followed by 4 h of antibiotic treatment (200 µg/mL gentamycin (Sigma Aldrich, St. Louis, MS, USA), 100 U/mL penicillin G (HighClone™)). LUG2012 infections were conducted at MOI 10 for 2 h followed by 4 h of antibiotic treatment (550 µg/mL gentamycin, 280 U/mL penicillin G, 280 µg/mL streptomycin, 5 U/mL lysostaphin (Sigma Aldrich)). Uninfected or rH1N1 infected cells served as controls. Controls received the same antibiotic treatment. After antibiotic treatment, cells were detached using a scraper and counted. The cell number was used for the normalization of oxylipin amounts (10 million cells per sample) for both absolute and relative quantified lipid mediators. The cells were harvested by centrifugation at 4 °C and the cell pellets were suspended in 500 µL ice-cold methanol supplemented with 0.1% BHT and 500 µL ice-cold water (HPLC–MS grade) and stored at −80 °C.

### 4.5. Oxylipin Extraction

The frozen cell pellets were thawed on ice and immediately transferred in a 2-mL tube containing FastPrep™ lysing matrix D and 100 µL internal standard was added. The cells were lysed for 45 s with 6 m/s using a FastPrep™. The supernatant was transferred into a new glass tube on ice. The lysing matrix was washed with 250 µL ice-cold water and 250 µL ice-cold methanol containing 0.1% BHT, then a second lysing cycle was performed and both supernatants were combined. Afterwards, an alkaline hydrolysis step and solid-phase extraction was performed as previously described [24].

### 4.6. HPLC-MS/MS Measurement

After drying under nitrogen flow (TurboVap^®^, Biotage^®^), the extract was reconstituted in 70 µL 80% ACN and measured (10 µL injection volume) with an Agilent^®^ HPLC system (1200 series), coupled to an Agilent^®^ 6460 Triple quadrupole mass spectrometer with electrospray ionization source in negative mode. The mobile phase was water with 0.05% acetic acid (A) and ACN (B). The gradient, column, and MS parameters are referred to in [24]. Using dynamic multiple reaction monitoring (MRM) measurements, the identification was done by standard compounds (MRM transitions, retention times, and optimized parameters can be found in Table S1).

### 4.7. Quantification and Statistics

Absolute quantification for HETEs and EETs was done using calibration curves (concentration range between 0.1 and 50 ng/sample; curve type quadratic, weighting 1/x) with MS-certified standards and 12-HETE-d8 as an internal standard. HEPE, HDHAs, and HODEs were normalized to the internal standard response (HEPE and HDHAs to 12-HETE-d8; HODEs to 13-HODE-d4) and stated as “relative amounts” in the plots. For absolute and relative quantified amounts of lipid mediators, the data were normalized to 10 million cells per sample. Data analysis was performed with Agilent Mass Hunter Qualitative and Quantitative Analysis software (version B.07.00 for both). Infection experiments were done with four biological replicates and 15 biological replicates for control (non-infection) experiments. Statistics were done using the Mann–Whitney U-test with Graph Pad Prism (version 7.05). The *p*-values were adjusted for multiple comparisons (control vs. three different mono-infections and two different co-infections) and the number of oxylipins using Bonferroni’s correction. The strength of the relationship between the measured lipid mediators was tested by correlation analysis after Spearman (ρ > 0.75) and resulted in the integration of all EETs and all HDHAs into two clusters under the inclusion of their biosynthesis. The clustering reduced the number of oxylipins used to calculate the factor of Bonferroni correction. The *p*-values obtained after correction were compared to a significance level α of 0.05. Both *p*-values and results from the correlation analysis can be found in Supplementary Materials (Tables S2 and S3).

## 5. Conclusions

In summary, the presented study demonstrates alterations in the oxylipin profile of 16HBE cells in response to different viral and bacterial mono and co-infections. The pro-inflammatory arachidonic acid metabolite 5-HETE was shown to have a prominent role for all analyzed conditions involving bacteria, which warrants further experimental studies to identify up-stream and down-stream mechanisms driving lipid mediator production.

## Figures and Tables

**Table 1:** Overview of measured lipid mediators with precursor and involved major enzymes. Enzyme abbreviations: lipoxygenase (LOX), cytochrome P450 (CYP), and cyclooxygenase (COX).

Precursor	Key Enzymes	Measured Lipid Mediators
arachidonic acid	5-LOX, 12-LOX, 15-LOX	5-HETE; 12-HETE; 15-HETE
	CYP ω-hydroxylase	20-HETE
	CYP epoxygenases	5,6-EET; 8,9-EET; 11,12-EET; 14,15-EET
linoleic acid	15-LOX	9-HODE, 13-HODE
docosahexaenoic acid	12-LOX; 15-LOX	13-HDHA, 14-HDHA, 17-HDHA
eicosapentaenoic acid	COX-2; CYP enzymes	18-HEPE

## Supplementary Materials

The following are available online at https://www.mdpi.com/2218-1989/10/3/113/s1, raw data. Figure S1: Survival rate of 16HBE cells under indicated infection conditions; 16HBE cells were infected as described in the Materials and Methods section; 6 h post bacterial infections, the vital cells were detached and counted; cell counts were normalized to the uninfected controls and are displayed as percentage of the uninfected control. Figure S2: Heatmap displaying fold changes (infection/control) of measured lipid mediators; decreased levels are shown in green and increased amounts in red. Table S1: Optimized MRM parameters for analyzed lipid mediators with qualifier^1^ and quantifier^2^ ion; grey lipid mediators were not detected. Table S2: Calculated *p*-values from Mann–Whitney test. Table S3: Spearman´s range correlation.

## Appendix A

Members of the KoInfekt Study Group: Dörte Becher (University of Greifswald), Barbara M. Bröker (University Medicine Greifswald), Sven Hammerschmidt (University of Greifswald), Lars Kaderali (University Medicine Greifswald), Bernd Kreikemeyer (University Medicine Rostock), Michael Lalk (University of Greifswald), Thomas C. Mettenleiter (Friedrich-Loeffler-Institute Isle of Riems), Brigitte Müller-Hilke (University Medicine Rostock), Katharina Riedel (University of Greifswald), Jochen Schubert (University Medicine Rostock), Ulrike Seifert (University Medicine Greifswald), Tim Urich (University of Greifswald), Uwe Völker (University Medicine Greifswald).
