# Supplementary material for: 16HBE Cell Lipid Mediator Responses to Mono and Co-Infections with Respiratory Pathogens
Source: Metabolites. 2020 Mar 18;10(3):113. doi: 10.3390/metabo10030113 (PMC7142531; doi:10.3390/metabo10030113)
Supplement: Supplementary file 1 [file metabolites-10-00113-s001.zip › Supplementary Materials/Supplementary Materials.pdf]

metabolites

Supplemental material

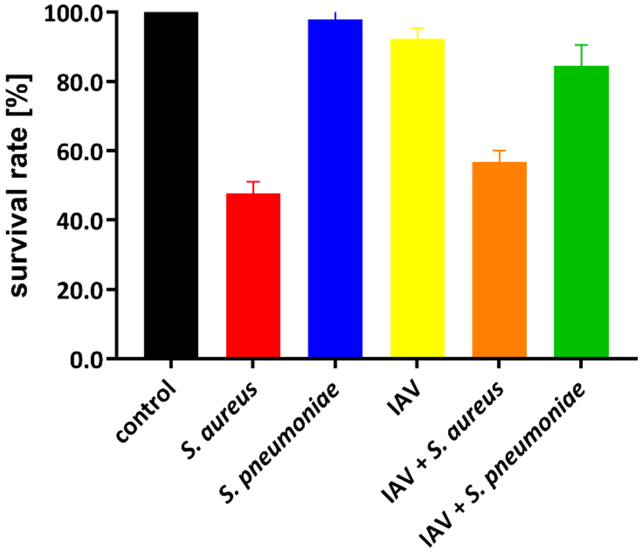

Figure S1. Survival rate of 16HBE cells under indicated infection conditions. 16HBE cells were infected (n=4) as described in the method section. 6 h post bacterial infections the vital cells were detached and counted. Cell counts were normalized to the uninfected controls (n=15) and are displayed as percentage of the uninfected control.

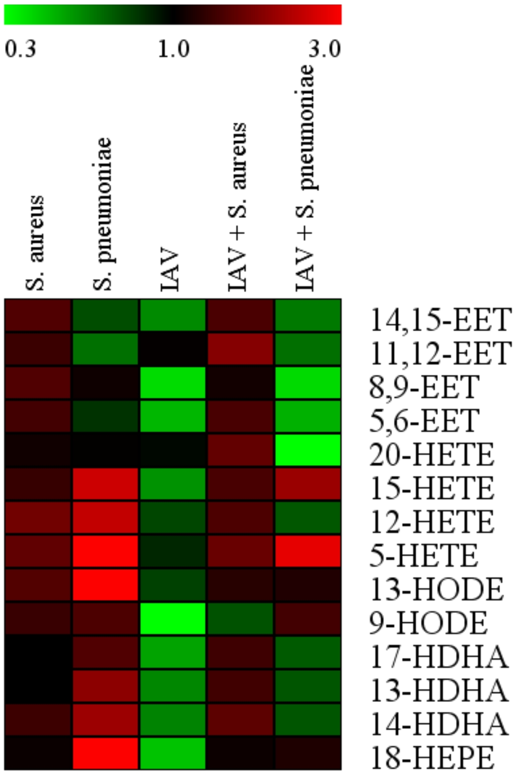

Figure S2. Heatmap displaying fold changes [infection (n=4)/control (n=15)] of measured lipid mediators. Decreased levels are shown in green and increased amounts in red.

Table S1. Optimized MRM parameters for analyzed lipid mediators with qualifier[1] and quantifier[2] ion. Grey lipid mediators were not detected.

compound	precursor ion	product ion	fragmentor voltage [V]	collision energy [V]	cell accelerator voltage [V]	retention time [min]
14,15-EET[1]	319.2	219.1	100	8	4	18.25
14,15-EET[2]	319.2	175.1	100	10	1	18.25
11,12-EET[1]	319.2	179.0	100	8	1	18.50
11,12-EET[2]	319.2	167.2	100	12	2	18.50
8,9-EET[1]	319.2	154.9	100	8	1	18.40
8,9-EET[2]	319.2	151.0	100	10	1	18.40
5,6-EET[1]	319.2	191.0	100	2	1	18.60
5,6-EET[2]	319.2	99.0	100	16	1	18.60
5,15-DiHETE[1]	335.3	255.3	100	12	3	15.50
5,15-DiHETE[2]	335.3	173.2	100	12	6	15.50
20-HETE[1]	319.2	288.9	140	12	2	16.90
20-HETE[2]	319.2	237.0	140	10	3	16.90
15-HETE[1]	319.2	219.2	120	4	4	17.45
15-HETE[2]	319.2	120.9	120	12	3	17.45
12-HETE[1]	319.2	163.0	120	12	1	17.70
12-HETE[2]	319.2	135.2	120	12	3	17.70
5-HETE[1]	319.2	203.3	100	12	5	17.80
13-HOTrE[1]	293.2	224.0	100	10	4	16.80
13-HOTrE[2]	293.2	210.8	100	6	4	16.80
13-HODE[1]	295.2	195.1	100	14	1	17.25
13-HODE[2]	295.2	277.2	100	10	2	17.25
9-HODE[1]	295.2	171.0	100	10	1	17.30
9-HODE[2]	295.2	123.1	100	14	3	17.30
17-HDHA[1]	343.2	245.1	100	8	3	17.50
17-HDHA[2]	343.2	201.1	100	10	4	17.50
14-HDHA[1]	343.2	234.1	100	10	4	17.60
14-HDHA[2]	343.2	161.2	100	8	1	17.60
13-HDHA[1]	343.2	193.1	80	10	3	17.65
13-HDHA[2]	343.2	221.0	80	12	5	17.65
18-HEPE[1]	317.2	215.0	80	10	4	16.75
18-HEPE[2]	317.2	259.0	80	6	3	16.75
15-HEPE[1]	317.2	255.3	100	4	2	17.00
15-HEPE[2]	317.2	219.2	100	4	4	17.00
Protectin DX[1]	359.2	206.1	100	12	4	15.70
Protectin DX[2]	359.2	153.1	100	12	1	15.70
Resolvin D5[1]	359.2	199.2	80	10	5	15.65
Resolvin D5[2]	359.2	141.0	80	10	2	15.65
Leukotriene B4	335.2	194.9	120	8	6	15.70
Leukotriene B4	335.2	58.9	120	6	3	15.70
Leukotriene E4	438.2	351.1	100	14	4	14.70
Leukotriene E4	438.2	333.0	100	12	5	14.70
12-HETE-d8	327.2	214.0	100	12	4	17.70
13-HODE-d4	299.3	198.2	100	14	6	17.25

Table S2. Calculated p-values from Mann Whitey test.

lipid mediator	group		reference	p value	adjusted p value
14,15-EET	*S. aureus*	vs.	control	0.0273	>0.9999
14,15-EET	*S. pneumoniae*	vs.	control	0.4107	>0.9999
14,15-EET	IAV	vs.	control	0.0366	>0.9999
14,15-EET	IAV + S. aureus	vs.	control	0.0062	0.279
14,15-EET	IAV + S. pneumoniae	vs.	control	0.1522	>0.9999
11,12-EET	*S. aureus*	vs.	control	0.0485	>0.9999
11,12-EET	*S. pneumoniae*	vs.	control	0.5304	>0.9999
11,12-EET	IAV	vs.	control	0.4107	>0.9999
11,12-EET	IAV + S. aureus	vs.	control	0.0093	0.4185
11,12-EET	IAV + S. pneumoniae	vs.	control	0.4107	>0.9999
8,9-EET	*S. aureus*	vs.	control	0.0093	0.4185
8,9-EET	*S. pneumoniae*	vs.	control	0.5304	>0.9999
8,9-EET	IAV	vs.	control	0.001	0.045
8,9-EET	IAV + S. aureus	vs.	control	0.5304	>0.9999
8,9-EET	IAV + S. pneumoniae	vs.	control	0.0062	0.279
5,6-EET	*S. aureus*	vs.	control	0.08	>0.9999
5,6-EET	*S. pneumoniae*	vs.	control	0.7363	>0.9999
5,6-EET	IAV	vs.	control	0.0196	0.882
5,6-EET	IAV + S. aureus	vs.	control	0.0624	>0.9999
5,6-EET	IAV + S. pneumoniae	vs.	control	0.0366	>0.9999
20-HETE	*S. aureus*	vs.	control	0.2208	>0.9999
20-HETE	*S. pneumoniae*	vs.	control	0.469	>0.9999
20-HETE	IAV	vs.	control	0.9613	>0.9999
20-HETE	IAV + S. aureus	vs.	control	0.08	>0.9999
20-HETE	IAV + S. pneumoniae	vs.	control	0.0005	0.0225
15-HETE	*S. aureus*	vs.	control	0.0366	>0.9999
15-HETE	*S. pneumoniae*	vs.	control	0.0005	0.0225
15-HETE	IAV	vs.	control	0.0196	0.882
15-HETE	IAV + S. aureus	vs.	control	0.0139	0.6255
15-HETE	IAV + S. pneumoniae	vs.	control	0.0021	0.0945
12-HETE	*S. aureus*	vs.	control	0.0005	0.0225
12-HETE	*S. pneumoniae*	vs.	control	0.0005	0.0225
12-HETE	IAV	vs.	control	0.2208	>0.9999
12-HETE	IAV + S. aureus	vs.	control	0.0093	0.4185
12-HETE	IAV + S. pneumoniae	vs.	control	0.1847	>0.9999
5-HETE	*S. aureus*	vs.	control	0.0005	0.0225
5-HETE	*S. pneumoniae*	vs.	control	0.0005	0.0225
5-HETE	IAV	vs.	control	0.4107	>0.9999
5-HETE	IAV + S. aureus	vs.	control	0.0005	0.0225
5-HETE	IAV + S. pneumoniae	vs.	control	0.0005	0.0225
13-HODE	*S. aureus*	vs.	control	0.08	>0.9999
13-HODE	*S. pneumoniae*	vs.	control	0.0005	0.0225

13-HODE	IAV	vs.	control	0.5304	>0.9999
13-HODE	IAV + S. aureus	vs.	control	0.307	>0.9999
13-HODE	IAV + S. pneumoniae	vs.	control	0.2208	>0.9999
9-HODE	*S. aureus*	vs.	control	0.2208	>0.9999
9-HODE	*S. pneumoniae*	vs.	control	0.0093	0.4185
9-HODE	IAV	vs.	control	0.001	0.045
9-HODE	IAV + S. aureus	vs.	control	0.3571	>0.9999
9-HODE	IAV + S. pneumoniae	vs.	control	0.307	>0.9999
17-HDHA	*S. aureus*	vs.	control	0.3571	>0.9999
17-HDHA	*S. pneumoniae*	vs.	control	0.0062	0.279
17-HDHA	IAV	vs.	control	0.0036	0.162
17-HDHA	IAV + S. aureus	vs.	control	0.0093	0.4185
17-HDHA	IAV + S. pneumoniae	vs.	control	0.0273	>0.9999
13-HDHA	*S. aureus*	vs.	control	>0.9999	>0.9999
13-HDHA	*S. pneumoniae*	vs.	control	0.0005	0.0225
13-HDHA	IAV	vs.	control	0.0273	>0.9999
13-HDHA	IAV + S. aureus	vs.	control	0.0366	>0.9999
13-HDHA	IAV + S. pneumoniae	vs.	control	0.1522	>0.9999
14-HDHA	*S. aureus*	vs.	control	0.156	>0.9999
14-HDHA	*S. pneumoniae*	vs.	control	0.0007	0.0315
14-HDHA	IAV	vs.	control	0.0464	>0.9999
14-HDHA	IAV + S. aureus	vs.	control	0.0118	0.531
14-HDHA	IAV + S. pneumoniae	vs.	control	0.1268	>0.9999
18-HEPE	*S. aureus*	vs.	control	0.9613	>0.9999
18-HEPE	*S. pneumoniae*	vs.	control	0.0005	0.0225
18-HEPE	IAV	vs.	control	0.0036	0.162
18-HEPE	IAV + S. aureus	vs.	control	0.5965	>0.9999
18-HEPE	IAV + S. pneumoniae	vs.	control	0.3571	>0.9999

Table S3. Spearman´s rang correlation

	14,15-EET	11,12-EET	8,9-EET	5,6-EET	20-HETE	15-HETE	12-HETE	5-HETE	13-HODE	9-HODE	17-HDHA	13-HDHA	14-HDHA	18-HEPE
14.15-EET		0.831	0.803	0.912	0.421	0.045	0.562	0.181	0.376	0.082	0.310	0.411	0.500	0.159
11.12-EET	0.831		0.553	0.794	0.490	-0.111	0.444	0.050	0.268	-0.288	0.075	0.248	0.317	-0.131
8.9-EET	0.803	0.553		0.894	0.485	0.133	0.714	0.223	0.395	0.222	0.496	0.627	0.717	0.418
5.6-EET	0.912	0.794	0.894		0.574	0.100	0.675	0.180	0.381	0.054	0.418	0.569	0.631	0.247
20-HETE	0.421	0.490	0.485	0.574		0.115	0.645	0.145	0.291	-0.203	0.530	0.642	0.638	0.199
15-HETE	0.045	-0.111	0.133	0.100	0.115		0.441	0.863	0.436	0.638	0.559	0.468	0.520	0.630
12-HETE	0.562	0.444	0.714	0.675	0.645	0.441		0.599	0.684	0.210	0.575	0.808	0.917	0.584
5-HETE	0.181	0.050	0.223	0.180	0.145	0.863	0.599		0.678	0.535	0.459	0.534	0.608	0.676
13-HODE	0.376	0.268	0.395	0.381	0.291	0.436	0.684	0.678		0.285	0.235	0.563	0.550	0.445
9-HODE	0.082	-0.288	0.222	0.054	-0.203	0.638	0.210	0.535	0.285		0.414	0.241	0.308	0.566
17-HDHA	0.310	0.075	0.496	0.418	0.530	0.559	0.575	0.459	0.235	0.414		0.830	0.795	0.715
13-HDHA	0.411	0.248	0.627	0.569	0.642	0.468	0.808	0.534	0.563	0.241	0.830		0.905	0.799
14-HDHA	0.500	0.317	0.717	0.631	0.638	0.520	0.917	0.608	0.550	0.308	0.795	0.905		0.727
18-HEPE	0.159	-0.131	0.418	0.247	0.199	0.630	0.584	0.676	0.445	0.566	0.715	0.799	0.727	
